# The effects of fermented vegetables on the gut microbiota for prevention of cardiovascular disease

**DOI:** 10.1017/gmb.2024.4

**Published:** 2024-05-09

**Authors:** Melissa Baron, Bin Zuo, Jianmin Chai, Jiangchao Zhao, Alireza Jahan-Mihan, Judy Ochrietor, Andrea Y. Arikawa

**Affiliations:** 1 Instructor of Nutrition and Dietetics, University of North Florida, Jacksonville, FL, USA; 2 Research Assistant of Animal Science, University of Arkansas, Fayetteville, AR, USA; 3 Schoo of Life Sciences, University of Foshan, Foshan, China; 4 Animal Science, University of Arkansas, Fayetteville, AR, USA; 5 Nutrition and Dietetics, University of North Florida, Jacksonville, FL, USA; 6 Biology, University of North Florida, Jacksonville, FL, USA

**Keywords:** gut microbiota, cardiovascular disease, fermented vegetables, inflammation, C-reactive protein

## Abstract

This study investigated the impact of regular consumption of fermented vegetables (FVs) on inflammation and the composition of the gut microbiota in adults at increased risk for cardiovascular disease. Eighty-seven adults ages 35–64 were randomized into an FV group, who consumed 100 g FVs daily at least five times per week for eight weeks, or a usual diet (UD) group. Blood and stool samples were obtained before and after the intervention. Dependent samples *t* tests and adjusted linear models were used for within- and between-group comparisons. The mean age and body mass index of participants were 45 years and 30 kg/m^2^, and 80% were female. Bloating or gas was the most common side effect reported (19.3% FV group vs. 9.4% UD group). There were no changes in C-reactive protein, oxidized low-density lipoprotein-receptor 1, angiopoietin-like protein 4, trimethylamine oxide, and lipopolysaccharide-binding protein or bacterial alpha diversity between groups. Our findings indicate that consuming 100 g of FVs for at least five days per week for eight weeks does not change inflammatory biomarkers or microbial alpha diversity as measured by the Shannon index. It is possible that higher doses of FVs are necessary to elicit a significant response by gut bacteria.

## Introduction

There is a large body of evidence documenting the role of the gut microbiota in health and disease. It has been reported that individuals diagnosed with coronary artery disease (CAD) have decreased diversity of the gut bacteria and increased richness of certain types of bacteria (Toya et al., 2020). It has also been suggested that the gut microbiota may be fostering inflammation by increasing the production of inflammatory molecules, therefore contributing to CVD (Tang et al., 2019). These findings suggest that the gut bacteria may play an important role in the development and progression of CVD. A recent case–control study found significantly decreased alpha diversity (*p* = 0.002), richness (*p* = .001), and evenness (*p* = 0.014) of gut bacteria in patients with advanced CAD (Toya et al., 2020). A cross-sectional study of 322 participants with large artery atherosclerotic stroke or transient ischaemic attack, and 231 healthy controls found that the control group had fewer opportunistic pathogens, including *Enterobacter*, *Megasphaera*, *Oscillibacter*, and *Desulfovibrio*, and more abundant beneficial bacteria: *Bacteroides*, *Prevotella*, and *Faecalibacterium* (Yin et al., 2015). Researchers have also identified microbial strains associated with atherosclerotic cardiovascular disease (ACVD) (Jie et al., 2017). When comparing 218 patients with ACVD and 187 healthy controls, a higher abundance of *Roseburia intestinalis* (a butyrate producing bacteria) and *Faecalibacterium cf. prausnitzii* was found in the control group, and a greater abundance of *Enterobacteriaceae* and *Streptococcus spp.* was noted in the participants with ACVD (Jie et al., 2017).

The gut microbiota plays a pivotal role in regulating inflammation through a multitude of mechanisms. These microorganisms are involved in maintaining intestinal barrier integrity, primarily by promoting the production of mucin and tight junction proteins. This barrier prevents the translocation of pathogenic microbes and their products into the systemic circulation, thereby averting immune responses. Additionally, the gut microbiota regulates the differentiation and function of immune cells, such as T cells, B cells, and macrophages, through direct interactions and metabolite production. Short-chain fatty acids (SCFAs), produced by certain gut bacteria during the fermentation of dietary fibre, exert anti-inflammatory effects by modulating immune cell responses and inhibiting pro-inflammatory cytokine production. Gut microbes can also metabolize dietary components into bioactive molecules that influence host immune and inflammatory responses. Dysbiosis or alterations in the composition of the gut microbiota can disrupt these regulatory mechanisms, contributing to chronic inflammation and various inflammatory diseases (Belkaid and Hand, 2014; Cani and Jordan, 2018). For example, a reduced or disrupted intestinal mucous layer thickness can increase the leakage of lipopolysaccharide (LPS), a component of the cell walls of Gram-negative bacteria, into the circulatory system. LPS activates the innate immune system, eliciting a low-grade systemic inflammatory response (Yücel et al., 2017), which is a key factor in triggering the onset of cardiovascular diseases (CVDs; Cani and Jordan, 2018). Trimethylamine oxide (TMAO) is another metabolite derived from the microbial metabolism of animal-derived foods which has been associated with an increased risk of CVD (Krueger et al., 2021) Furthermore, gut microbiota metabolites have also been implicated in changes in the activity of angiopoietin-like protein 4, which disrupt lipoprotein lipase activity and lead to lipid dysregulation, thus increasing the risk of CVD (Zwartjes et al., 2021). Finally, certain microbial taxa have been associated with low-grade inflammation assessed via high sensitivity C-reactive protein (hsCRP) and LPS levels (van den Munckhof et al., 2018). *Bifidobacterium* abundance has been associated with lower hsCRP and LPS levels, and *Faecalibacterium prausnitzii* abundance has been associated with lower hsCRP levels. Conversely, lower abundance of *Ruminococcaceae* and *Ruminococcus* have been associated with higher levels of hsCRP (van den Munckhof et al., 2018).

Among the known risk factors for CVD, diet is arguably the most significant because it not only contributes to other risk factors, such as high blood pressure, obesity, inflammation, high blood lipids, and diabetes, but also decreases the risk for CVD through delivery of protective nutrients. Fermented vegetables (FVs) contain live probiotic bacteria that are associated with health benefits, but only a few human studies have evaluated the effects of consumption of FVs on changes in gut microbiota and their possible impact on human health. There is very limited prior research investigating FV consumption in the context of CVD and the gut microbiota. The purpose of this study was to investigate the impact of regular consumption of FVs on markers of inflammation and the composition of the gut microbiota in adults at increased risk for CVD. We hypothesized that regular consumption of FVs would improve both the biomarkers of inflammation and the composition of the gut microbiota in adults at risk for CVD.

## Methods

### Study design and participants

**Figure 1.**
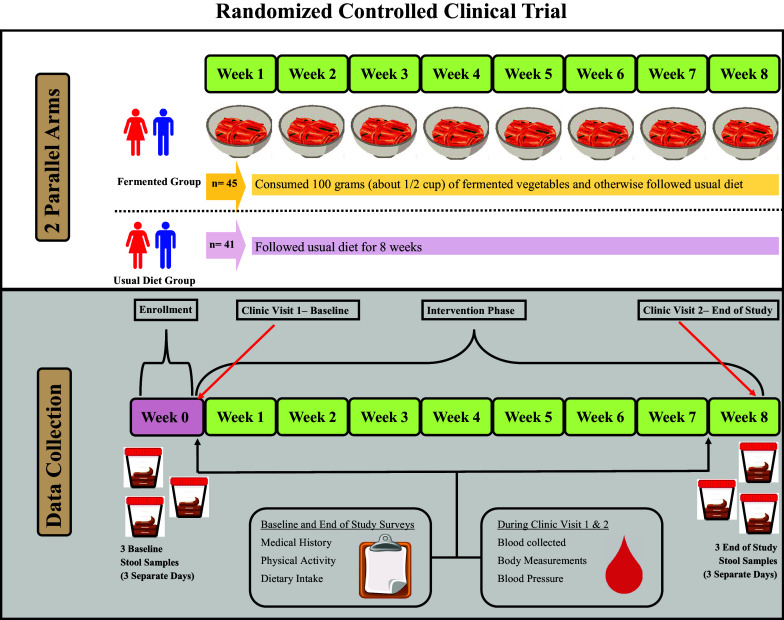
Diagram of the study design. This randomized controlled trial consisted of two parallel groups, the fermented vegetable group consumed 100 *g* of fermented vegetables once daily for 8 weeks and the usual diet group consumed their usual diet for 8 weeks. Stool and blood samples were collected prior to randomization and at the end of the 8-week period. Participants also filled out questionnaires twice during the study.

This study was a randomized controlled clinical trial with two parallel groups, an FV group and a usual diet (UD) group (clinicaltrials.gov ID: NCT04887662). Participants in the UD group followed their UD while participants in the FV group consumed 100 grams of FVs five days a week and otherwise followed their UD. Figure 1 shows the study design, and Table 1 shows participant inclusion and exclusion criteria. The target population was men and women aged 35–64 years who had at least one risk factor for CVD. This study aimed to recruit between 90 and 100 participants with the goal of having 80 participants complete the study (a 20% dropout rate was assumed). The sample size was calculated using C-reactive protein (CRP) data from a recently completed pilot study (Galena et al., 2022). The following parameters were used for the sample size calculation: mean CRP levels = 176 ng/ml, standard deviation = 100 ng/ml, power = 85%, and alpha = 0.05. Based on these parameters, the target sample size was 37 participants per group.

**Table 1.** Inclusion and exclusion criteria for study participants

Inclusion criteria
Age 35–64 Participants must meet at least one of the following criteria: Overweight or obese (determined by BMI >25), Family history of heart disease, controlled hypertension Willing to consume one half cup of fermented vegetables 5 days a week for 8 weeks No disabilities that may limit capacity to provide informed consent Willing to visit the clinic twice during an 8–week period for body measurements and blood draws A signed informed consent Not on statin (Lipitor, Lesol, Crestor, Zocor, Altoprev) Not on medication for diabetes (insulin, metformin, glipizide, glimepiride, glyburide) Not on the following Monoamine oxidase inhibitors (Emsam, Marplan, Nardil, Parnate) Not on antirheumatic medications Not on biologics (Humira, Enbrel, Orencia, Kineret)
Exclusion criteria
Regular consumption of probiotics Regular consumption of fermented vegetables (two times per week or more) Smoker Previous or current diagnosis of cancer Diagnosis of diabetes mellitus Diagnosis of Crohn’s disease Diagnosis of colitis Diagnosis of rheumatoid arthritis Diagnosis of psoriasis Taking antibiotics during the past three months

This project was approved by the University of North Florida (UNF) Institutional Review Board (IRB) and all participants provided informed consent prior to starting the study (UNF IRB Number: 1712254-1).

### Treatment groups

Participants in the FV group were instructed to consume 100 grams of FVs at least 5 out of 7 days each week for eight weeks. There were no restrictions on the timing and composition of meals consumed along with the FVs. However, participants were instructed by the study registered dietician to not cook or heat the FVs and to reduce their sodium intake to account for the extra 600 mg of sodium consumed daily via the FVs. The UD group was asked to consume their current diet and not make any changes.

Throughout the study, FVs were delivered weekly by a local producer specialized in fermented krauts and kimchi. Eight varieties of kraut and kimchi were stocked in the study kitchen, and participants in the FV group selected their preferred varieties. Approximately 2.7 kg of FVs (about 27 100 g servings) were provided to the participants randomized into the FV group at their first clinic visit. At the midpoint of the study, these participants were contacted to plan for delivery or pickup of an additional 2.7 kg of FVs.

### Production of FVs

The FVs used in the study were made by a local producer through natural fermentation. The process starts by combining chopped cabbage with brine (2% sea salt) in a large wooden barrel as well as spices and condiments. Under anaerobic conditions, and at a temperature of approximately 23 °C, the bacteria that are naturally present in the cabbage start to grow while producing lactic acid, which lowers the pH of the environment and prevents growth of other bacteria that may be harmful to health. It takes approximately 4–5 days for the finished product to be ready for consumption, which is determined based on measurement of the salinity and pH of the product as well as on the taste, texture, and colour. Once the FVs reach the desired sensory characteristics, they are stored at 4 °C for up to 6 months. All FVs provided were produced within one month of delivery to the study participants. Our laboratory has previously analysed the microbial composition of samples of fermented cabbage and the analysis indicates the predominance of two major genera, namely *Lactobacillus* and *Leuconostoc*, which is consistent with previous reports (Zabat et al., 2018; Park et al., 2021). It is well established that once the pH of fermented cabbage reaches 3.5, the presence of bacteria is restricted to the genera mentioned above (Zabat et al., 2018).

### Study procedures

Participants were recruited through many strategies: printed flyers in high-visibility areas, a study website, social media, recruitment emails, and word of mouth. Interested participants were directed to the study website to fill out a screening questionnaire. Participants who met the eligibility requirements were emailed a link to an orientation video and a consent form for their review. Upon consent to participate in the study, participants scheduled an appointment time for the initial clinic visit.

Participants attended a clinic visit twice during the study: at 0 weeks (initial clinic visit) and at 8 weeks (final clinic visit) where blood was drawn, and blood pressure and body composition were assessed. Additionally, participants completed online surveys to assess medical history, physical activity level, demographics, and prescription medication use. All participants were monitored through online weekly gastrointestinal symptom logs throughout the study period.

### Data collection

#### Surveys

The DHQ-3, (*Diet History Questionnaire III (DHQ III) | EGRP/DCCPS/NCI/NIH*, n.d.) 199, a 135-item food frequency questionnaire designed by the National Cancer Institute, was used to assess the participants’ diet intake prior to the clinic visits. A 24-Hour Recall questionnaire was used to assess dietary intake at three time points: baseline (0 week), mid-study (4 weeks), and at end of study (8 weeks). Participants were also asked to complete eight weekly symptom logs to record their weekly vegetable intake and any symptoms they may have had related to gastrointestinal function including frequency of defecation, bloating or gas, abdominal pain, diarrhoea/very loose stools, swelling in hands, legs or feet, and any other symptoms they chose to report.

#### Stool collection

Participants received stool collection kits prior to their clinic visits and were instructed to collect three stool samples on three separate days at two time points (weeks 0 and 8). Each stool collection kit contained three flushable stool collection sheets, three DNA/RNA Shield Faecal Collection tubes (Zymo Research, Irvine, CA) one biohazard bag, and detailed instructions for stool collection. Participants were asked to record the date and time of stool collection on each tube and store the tubes at room temperature until their clinic visit appointments. The time between collection of stool samples and the clinic visits ranged between one and six days.

#### Clinical data

All biological samples were processed and stored at −70C until analysis. Blood was collected in two 8-mL tubes and left at room temperature for 30 minutes before centrifugation at 25C for 10 minutes at 1400 rpm. Serum was transferred to 1.5 mL cryogenic tubes in 1-mL aliquots. Stool samples were kept in their original collection tubes and stored at −70C until analysis. The study staff obtained participants’ height, weight, and body composition at each clinic visit. A Detecto 439 Eye Level Beam Physician Scale 400ib *×* 4 oz with height rod was used to measure height in centimetres. Weight and percent body fat were measured by multifrequency bioelectrical impedance (InBody 570, Cerritos, CA). Blood pressure was measured by a digital blood pressure monitor (OMRON Model: HEM-907XL).

### Measurement of biomarkers

Biomarkers related to CVD and inflammation were selected based on other studies, particularly if there was a connection to the gut microbiome. Serum CRP (RandD Systems, Minneapolis, MN – Cat#DCRP00), angiopoietin-like protein 4 (ANGPTL4) (RandD Systems, Minneapolis, MN – Cat#DY3485), oxidized low-density lipoprotein (LDL) receptor 1 (LOX-1) (RandD Systems, Minneapolis, MN – Cat#DY1798), and TMAO (AFG Bioscience, Northbrook, IL – Cat#EK715704) were measured with commercial ELISA kits. Serum lipopolysaccharide-binding protein (LBP) was measured by a Pierce LAL chromogenic endotoxin quantitation kit (Cat#88282, ThermoFisher Scientific, Waltham, MA). All analyses were conducted in the laboratory of Dr. Arikawa at the UNF.

### 16S rRNA sequencing

DNA was extracted from the frozen stool samples with the DNeasy PowerLyzer PowerSoil Kit (Qiagen, Germantown, MD, USA) according to the manufacturer’s protocol. A NanoDrop One (Thermo Fisher Scientific, Madison, WI, USA) was used to measure DNA concentration and diluted to 10 ng/μL. Next-generation sequencing of the V4 region of the 16S rRNA gene was performed. Amplicon PCR was performed on the V4 region of 16S rRNA using the forward (5′-GTGCCAGCMGCCGCGGTAA-3′) and reverse (5′-GACTACHVGGGTWTCTAAT-3′) primers. PCR amplicons were barcoded and pooled in equal concentrations using the SequalPrep Normalization Plate Kit (Invitrogen, Carlsbad, CA, USA). qPCR was used to quantify and consolidate libraries using the Kappa Library Quantification Kit (Roche, Indianapolis, IN, USA), and the quality of the library will be determined by an Agilent 2100 Bioanalyzer (Agilent, Santa Clara, CA, USA). Positive and negative controls were sequenced for quality control. The ZymoBIOMICS™ Microbial Community Standard (Zymo Research, Irvine, CA, USA) was used to provide a commercial community DNA for a positive control, and DNA extraction and PCR amplification provided the negative controls. Sequencing was performed in a pair-end modality on the Illumina MiSeq 500 platform rendering 2 *×* 150 bp paired-end sequences (Illumina, San Diego, CA, USA). Sequencing reads after quality control were denoised using Deblur integrated with QIIME2 (2022.02 released), alignment against a 16S reference database (SILVA v132), and clustering into amplicon sequence variants (ASVs) with a 100% identity threshold. A total of 515 faecal samples were extracted for DNA and processed into the QIIME2 pipeline. After filtering and denoising, 484 samples were retained for microbiome analysis.

### Statistical data analysis

Descriptive statistics were obtained for each group (FV and UD) by calculating frequencies and percentages for categorical variables and means and standard deviation for continuous variables.

Comparisons were made between groups and within groups for all main and secondary outcomes. The independent variable of interest was consumption of FVs.

Analysis of covariance was used to compare the two groups with respect to blood biomarkers after controlling for sex, age at baseline, and body mass index (BMI). All *p*-values lower than 0.05 were considered statistically significant. Pearson’s correlation coefficients were calculated between all outcomes and alterations in the intestinal microbiota. IBM SPSS (Statistical Package for the Social Sciences) version 27 was used to perform statistical analysis.

Alpha diversity (microbial diversity within each sample) and beta diversity (microbial diversity between samples) were calculated using the q2-diversity plugin in QIIME2 at the ASV level. For alpha diversity, we used observed ASVs to measure microbial richness (number of species present) and the Shannon index to measure species richness and evenness (distribution). As for beta diversity, principal component analysis (PCoA) was used to discover the percent of variability and potential associations among the groups represented by the Bray–Curtis (measure of differences in taxa abundance between communities) and Jaccard index (taxa presence/absence). An analysis of similarity (ANOSIM) was used to assess significant clustering differences by comparing within-and between-group similarity. Finally, linear discriminant analysis (LDA) effect size (LEfSe) was used to identify specific bacterial features that were enriched between conditions and diet patterns in each group or subgroup at the ASV level. The LEfSe analysis was performed using a web-based tool with 0.05 as the alpha value for the pairwise Wilcoxon test, and 2.00 as the threshold on the LDA score. All microbial analyses were conducted in the laboratory of Dr. Zhao in the Department of Animal Science at the University of Arkansas.

## Results

**Figure 2.**
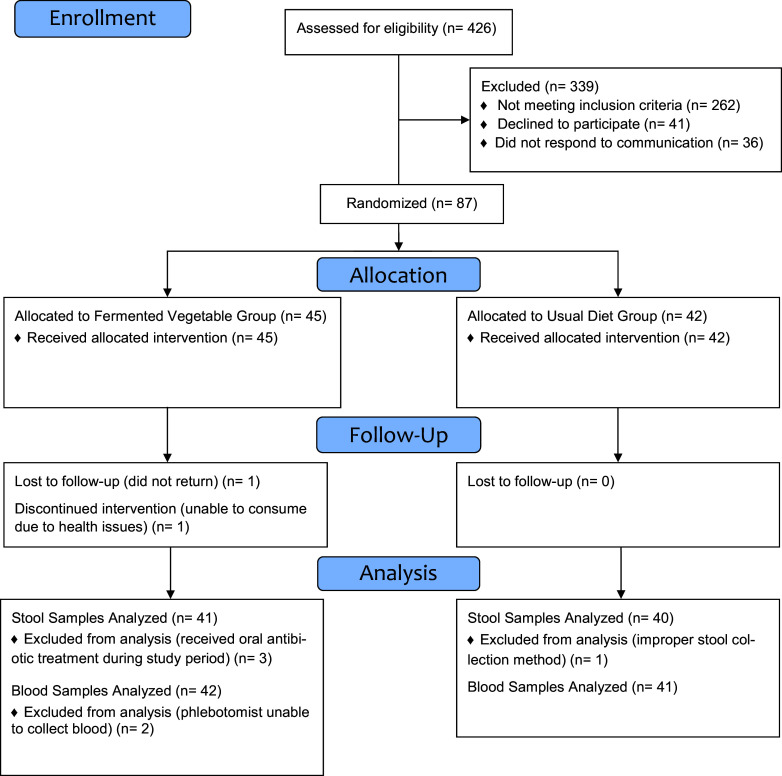
Flow of participants.

Recruitment, screening, and enrolment occurred between May and September 2021 and the study was completed by the end of November 2021. Figure 2 shows the participant flow through the study. Out of 426 potential participants who were screened prior to eligibility assessment, 87 participants were randomized into one of the two treatment groups and 86 completed the study. All study aims were assessed in at least 40 participants from each group. The most common reasons for being excluded from the study were not meeting either the age criteria or not presenting with a cardiovascular risk factor criterion.

The demographic characteristics of the study population stratified by study group are shown in Table 2. The two groups were similar in baseline characteristics. Approximately 80% and 78% of the participants in the UD group (*N* = 33) and FV group (*N* = 35) were female, respectively. The average age was 45 years for both study groups. BMI was 30.0 for both study groups. Most participants identified as white (81% and 85% in the UD and FV groups, respectively). There were no differences between groups in baseline nutrient intake (Table 2). The FV group showed a significant decrease in total calories and sodium intake at the end of the study compared with baseline, indicating that participants followed the instructions from the dietician to reduce sodium consumption during the study.

**Table 2.** Dietary variables before and after the study by treatment group.

	Mean (standard error)		
Variables	Before	After	*P*-value^a^
Calories (kcal)			
Usual diet	1982 (104)	2025 (116)	0.992
Fermented vegetable	2092 (106)	1807 (93.7)	**0.025**
*P*–value^b^	0.462	0.146	
Carbohydrates (g)			
Usual diet	213.0 (15.0)	220.4 (14.9)	0.845
Fermented VEGETABLE	206.5 (12.3)	182.7 (11.0)	0.589
*P*–value^b^	0.191	0.148	
Protein (g)			
Usual diet	80.1 (5.4)	81.5 (5.2)	0.478
Fermented vegetable	96.2 (7.2)	80.0 (5.9)	0.177
*P*–value^b^	0.096	0.186	
Fat (g)			
Usual diet	87.4 (5.8)	90.2 (6.1)	0.885
Fermented vegetable	92.9 (5.9)	81.3 (5.1)	0.184
*P*–value^b^	0.986	0.689	
Fibre (g)			
Usual diet	19.1 (1.8)	21.6 (1.8)	0.376
Fermented vegetable	20.2 (1.6)	18.3 (1.4)	0.271
*P*–value^b^	0.886	0.453	
Cholesterol (mg)			
Usual diet	353.0 (41.6)	280.9 (28.7)	0.428
Fermented vegetable	385.5 (57.0)	278.9 (32.8)	0.878
*P*–value^b^	0.939	0.497	
Sodium (g)			
Usual diet	3.2 (0.2)	3.3 (0.2)	0.228
Fermented vegetable	3.5 (0.2)	3.0 (0.2)	**0.048**
*P*–value^b^	0.449	0.977	
Alcohol (g)			
Usual diet	7.8 (2.2)	5.4 (1.9)	0.601
Fermented vegetable	9.8 (2.8)	7.4 (2.3)	0.165
*P*–value^b^	0.629	0.438	
Choline (g)			
Usual diet	374.6 (32.1)	325.2 (22.8)	0.364
Fermented vegetable	401.9 (40.8)	307.1 (27.0)	0.846
*P*–value^b^	0.938	0.732	

*Note*: (*N* = 42, usual diet and *N* = 44, fermented vegetable).

*P*-values lower than 0.05 are shown in bold.

a *P*-value for within-group comparisons using a dependent samples *t* test.

b *P*-value for between-group comparisons using linear models adjusted for total calorie intake.

There were no significant differences in either systolic or diastolic blood pressure among the two study groups at baseline and after the intervention (Table 3). Additionally, there were no significant differences in any of the measured biomarkers between or within study groups (Table 2).

**Table 3.** Demographic and baseline characteristics of study participants^a^

Characteristics	Usual diet (*n* = 42)	Fermented vegetable (*n* = 44)
Age at baseline (y)	45.4 (7.6)	44.8 (7.0)
Sex		
Male	8 (19.5)	10 (22.2)
Female	33 (80.5)	35 (77.8)
Race		
White	33 (80.5)	39 (86.7)
Non–White	7 (17.1)	6 (13.3)
Do not wish to provide	1 (2.4)	–
Ethnicity		
Hispanic	4 (9.8)	2 (4.4)
Non–Hispanic	37 (90.2)	42 (93.3)
Percent body fat	36.7 (9.7)	35.9 (9.1)
BMI (baseline)	30.0 (6.0)	30.0 (6.4)
Systolic BP (baseline)	120.7 (2.3)	122.1 (2.2)
Diastolic BP (baseline)	80.0 (1.5)	80.2 (1.4)

a Values are expressed as mean (standard deviation) for continuous variables and Frequency (%) for categorical variables.

The most common side effect reported was bloating or gas (9.4% in the UD group and 19.3% in the FV group) (Table 4). Average intake of FVs was 630 g per week. Average bowel movement frequency was 0.8 times per day in the UD group, and 1.9 times per day in the FV group.

**Table 4.** Frequency (%) of reported side effects by treatment group

Side effect	Usual diet (*n* = 42)	Fermented vegetable (*n* = 44)
Bloating or gas	9.4%	19.3%
Abdominal pain	2.7%	6.4%
Nausea	1.6%	4.1%
Diarrhoea or loose stool	0.2%	1%
Swelling of hands, feet, arms, or legs	0.8%	3.2%

### Changes in inflammatory biomarkers

There were no significant changes in blood levels of TMAO, LBP, CRP, ANGPTL4, and LOX-1 in the FV group compared with the UD group following consumption of 100 grams of FVs at least five days per week for eight weeks (Table **5**).

**Table 5.** Changes in inflammatory biomarkers by treatment group^a^

	Treatment groups		
Variables	Usual diet	Fermented vegetable	*P*-value^b^
TMAO (ng/mL)			
Week 0	479.8 (47.9)	360.3 (45.6)	0.425
Week 8	455.5 (43.9)	351.0 (41.8)	0.507
*P–*value^c^	0.059	0.661	
TMAO change	−24.3 (11.3)	−9.3 (10.8)	0.339
LBP (ng/mL)			
Week 0	22.9 (1.2)	23.9 (1.2)	0.793
Week 8	24.5 (1.3)	22.5 (1.3)	0.651
*P–*value^c^	0.751	0.646	
LBP change	0.6 (1.8)	−0.4 (1.7)	0.696
CRP (ng/mL)			
Week 0	7557.9 (2561.6)	5443.8 (2501.2)	0.882
Week 8	8053.9 (2646.1)	6086.6 (2583.8)	0.863
*P–*value^c^	0.848	0.456	
CRP change	569.2 (894.6)	1008.5 (831.2)	0.72
			
ANGLPT4 (ng/mL)			
Week 0	136.2 (25.0)	120.2 (25.3)	0.868
Week 8	132.1 (23.3)	120.7 (23.6)	0.859
*P–*value^c^	0.426	0.991	
ANGLPT4 change	0.05 (4.7)	−3.7 (4.6)	0.568
			
LOX–1 (pg/mL)			
Week 0	42.3 (13.4)	87.7 (35.2)	0.317
Week 8	40.6 (93.7)	80.9 (217.1)	0.36
*P–*value^c^	0.302	0.07	
OLR1 change	−5.9 (27.5)	−8.3 (26.6)	0.949

Abbreviations: ANGPTL4, angiopoietin-like 4; CRP, C-reactive protein; LBP, lipopolysaccharide-binding protein; LOX-1, oxidized LDL receptor 1; TMAO, trimethylamine oxide.

a Values are expressed as mean (standard error)

UD group, *N* = 40; FV group, *N* = 44.

b *P*-values for between-group comparisons, using analysis of covariance, adjusting for sex, age, and BMI.

c *P*-values for within-group comparisons via dependent samples *t* tests.

### Alpha and beta diversity

**Figure 3.**
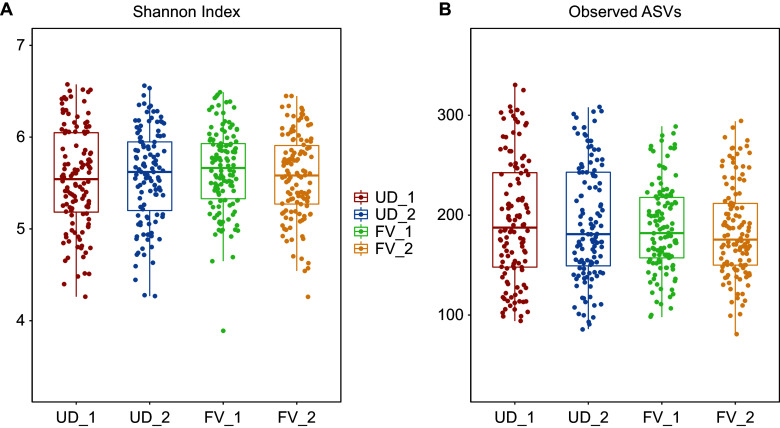
Microbial diversity expressed as Shannon index and observed amplicon sequence variants (ASVs) by treatment group and time point. Shannon index was calculated on the ASV level. UD_1 = usual diet group at time point 1, UD_2 = usual diet group at time point 2, FV_1 = fermented vegetable group at time point 1, and FV_2 = fermented vegetable group at time point 2.

Figure 3 portrays the box plots of both Shannon index and observed ASVs by treatment group and study time point. No significant differences in alpha diversity were observed between or within groups.

**Figure 4.**
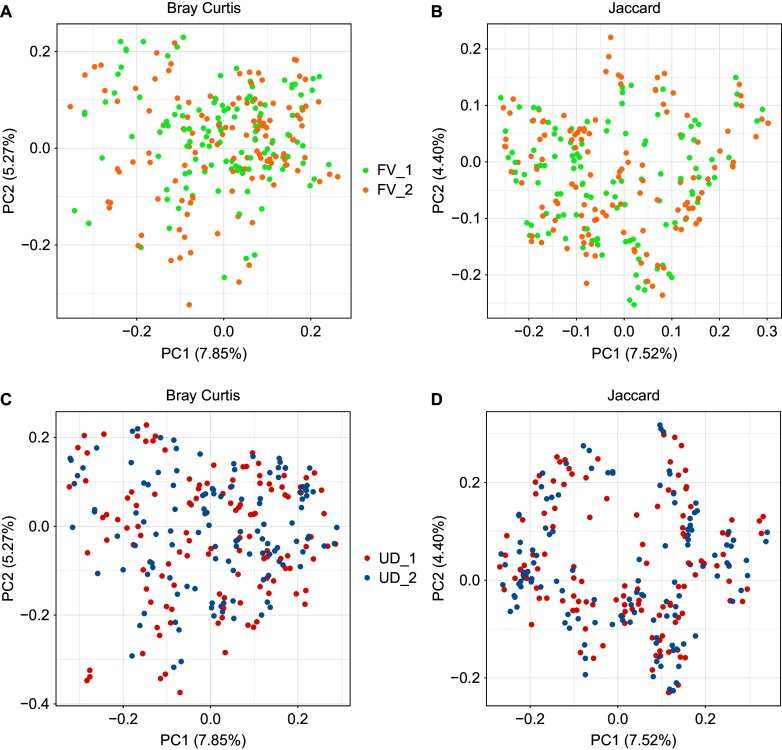
Microbial β-diversity plots expressed as Bray–Curtis and Jaccard distances by treatment group and study time point. The distances were calculated on the ASV level. UD_1 = usual diet group at time point 1, UD_2 = usual diet group at time point 2, FV_1 = fermented vegetable group at time point 1, and FV_2 = fermented vegetable group at time point 2.

PCoA plots of the Bray–Curtis and Jaccard indices are shown in Figure 4. Significant dissimilarities in beta diversity were seen between the FV and UD groups at both time points (*P* = 0.004); however, dissimilarities were not seen within each group over time. We also plotted weighted and unweighted Unifrac distances, and even though there was an increase in the proportion of the PC axis explained by the treatments, there were no significant differences between the two time points according to analysis of similarities (Supplementary Figure S1).

### LDA effect size

**Figure 5.**
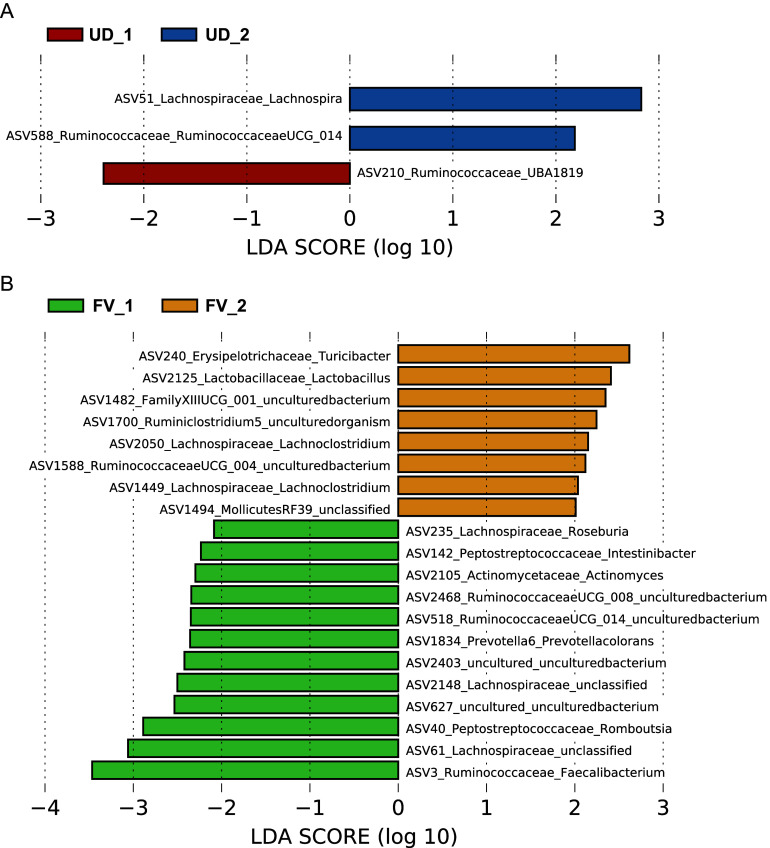
Within-group linear discriminant analysis effect size on ASV level. UD_1 = usual diet group at time point 1, UD_2 = usual diet group at time point 2, FV_1 = fermented vegetable group at time point 1, and FV_2 = fermented vegetable group at time point 2.

LDA effect size (LEfSe) was utilized to identify the bacterial taxa characterizing the differences between and within groups as shown in Figure 5. The FV group showed the most change in taxa between the two time points. Some notable changes were an increase in relative abundance of *Lactobacillaceeae Lactobacillus*, and *Lachnospiraceae Lachnoclostridium.* There was also a decrease in *Rumunococcaceae Faecalibacterium*, *Prevotella Prevotellacolorans*, *Actinomycetaceae Actinomyces*, *Peptostreptococcaceae Intestinobacter*, and *Lachnospiraceae Roseburia* in the intervention group.

**Figure 6.**
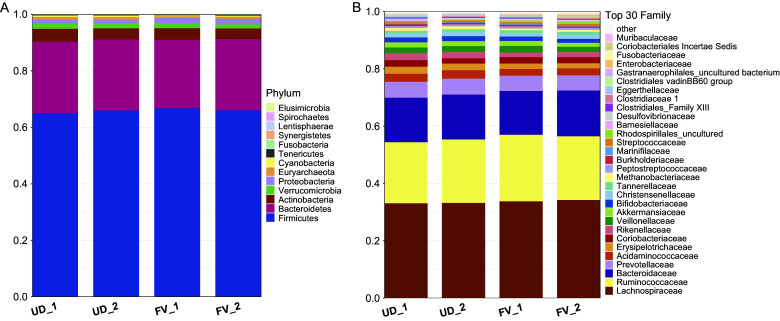
Microbial composition at the phylum and family levels ranked by relative abundance per treatment group and time point. UD_1 = usual diet group at time point 1, UD_2 = usual diet group at time point 2, FV_1 = fermented vegetable group at time point 1, and FV_2 = fermented vegetable group at time point 2.

The top three predominant phyla in the stool samples of the study participants were Firmicutes Bacteroidetes, and Actinobacter. There were no significant differences within or between groups in the relative abundance of the top phyla or top 15 families, as shown in Figure 6.

## Discussion

This randomized controlled trial explored the impact of regular consumption of FVs on markers of inflammation and the composition of the gut microbiota in adult men and woman at increased risk for CVD.

### Effect on inflammatory markers

There was no effect of FVs on serum biomarkers. Our results were similar to those of a pilot study conducted by our laboratory in which 31 females consumed 100 grams of fermented cucumbers or cabbage daily for six weeks (Galena et al., 2022). CRP, tumour necrosis factor alpha, and LBP were measured, and no significant changes were found between the FV and the non-FV groups. In turn, Wastyk *et al.* found decreased inflammatory markers in response to a 10-week dietary intervention of fermented foods in healthy adults (Wastyk et al., 2021). Circulating cytokines, chemokines, and inflammatory serum proteins were measured in the serum, and 19 of the 93 inflammatory markers decreased in the group consuming an average of 6.3 servings of fermented foods per day. Another study investigated how total antioxidant status (TAS) and serum lipids were influenced by kimchi consumption (Choi et al., 2013). In this seven-day study, 100 participants living in South Korea were recruited and assigned to either the low kimchi group (15 grams/day), or the high kimchi group (210 grams/day). TAS increased significantly in both groups (*p* < .001), and the greatest improvement was observed in the higher dose kimchi group (7.5% increase in the high kimchi group and 5.1% increase in the low kimchi group). Additionally, LDL and fasting blood glucose (FBG) were lower in the high kimchi group when compared with the low kimchi intake group. Fasting blood glucose (FBG) was also reduced in the high kimchi intake group (*p* = 0.003) (Choi et al., 2013). Consumption of 210 *g* per day of kimchi for 12 weeks has also been shown to improve inflammatory markers and the composition of the microbiome in individuals suffering from irritable bowel syndrome, in a recent randomized controlled trial (Kim et al., 2022), indicating that FV intake can be a viable treatment for patients affected by gut dysbiosis. The findings from these studies suggest that the dose and duration of intake of FVs may be important to alter levels of inflammatory markers, and the 100 g utilized in the present study may not have been sufficient to elicit changes in these markers.

To our knowledge, this was the first study to investigate the role of FVs on oxidized LDL receptor. Various dietary interventions have previously been shown to reduce oxidized LDL levels in different populations. Adherence to a 12-week Mediterranean dietary pattern significantly decreased oxidized LDL by 11/3% in 71 healthy women (Lapointe et al., 2005). In addition, there is compelling evidence from in vitro studies to clinical trials reporting on the benefits of antioxidant supplementation to reduce LDL oxidation (Kiokias et al., 2018). While FV intake did not change the levels of oxidized LDL receptor in the present study, it is possible that the dose provided was not sufficient to result in positive changes in lipid metabolism. Similarly, TMAO levels were not affected by consumption of FVs in this study. Indeed, the relationship between TMAO production, diet, and the gut microbiota is complex, and it has been hypothesized that TMAO may have different functions depending on the presence or absence of metabolic disease (Krueger et al., 2021). Previous studies have found that carnitine supplementation significantly increases TMAO levels in plasma (Samulak et al., 2019; Wu et al., 2019), while consumption of plant-based foods is associated with lower TMAO levels (Krueger et al., 2021). Wu et al. (2019) found that omnivores and vegetarians have distinct patterns of TMAO production and that omnivores had 10 times higher odds of being high TMAO producers compared with vegetarians. In addition, it has been reported that high TMAO producers have a higher Firmicutes to Bacteroidetes ratio compared with low producers (Wu et al., 2019). Li et al. (2022) showed that the ability to produce TMAO following the intake of animal-derived foods was dependent on a microbial profile that included species that predicted TMAO concentrations, such as *Eubacterium hallii*, *Eubacterium biforme*, *Roseburia hominis*, and *Alistipes shahii.* There was no evidence that FV consumption in the present study altered any of the TMAO-predicting species aforementioned, which may be one reason why there were no significant changes in plasma TMAO levels. We also examined red meat, choline, and animal protein intake in the study participants (data not shown) and did not find any significant differences between or within groups. The current published evidence on the factors associated with TMAO production indicates that if consumption of FVs can modulate TMAO levels, it is likely to occur through more pronounced changes in intake of other dietary components, such as red meat and plant-based foods, and, most importantly, carefully considering the composition of the gut bacteria to focus on species that can predict TMAO production.

### Effect on gut microbiota

Overall, we found slight changes in the gut microbiota profile in participants who consumed FVs; however, there was no effect on alpha or beta diversity. Significant differences in beta diversity (Bray–Curtis) were seen between the FV and UD groups at both time points (*p* = 0.004); however, there were no within-group changes, indicating that the dissimilarities were not related to the intervention. To further investigate whether specific bacterial taxa changed within each group during the 8-week intervention, LDA LEfSe was utilized. Eight taxa increased and 12 decreased in the FV group, and two increased and one decreased in the UD group.

*Lactobacillaceae Lactobacillus* was enriched in the final stool samples of only the FV group. This may be related to the content of FVs, as *Lactobacillus* is commonly found in fermented foods like kimchi and sauerkraut. Interestingly, *Lactobacillaceae Lactobacillus* was not enriched in our pilot study where participants consumed the same amount of FVs (Galena et al., 2022). The increase in *Lactobacillaceae Lactobacillus* in the present study may be related to the longer study period of 8 weeks, compared to 6 weeks in the pilot study. Many studies on probiotics have examined the effects of *Lactobacillus* with positive outcomes (Azad et al., 2018; Heeney et al., 2018; Tomova et al., 2019). Individuals with a lower abundance of *Lactobacillus* may suffer from constipation (Wang and Yao, 2021). Interestingly, participants in the FV group in the present study reported more frequent bowel movements compared with the UD group. Enrichment of *Lactobacillus* is also positively associated with increased abundance of both beneficial SCFAs such as butyrate, and saturated long-chain fatty acid which are thought to enhance gastrointestinal motility (Zhao et al., 2018). Conversely, our findings also indicated a lower abundance of the genus *Faecalibacterium* in the FV group at the end of the study, which are well-known butyrate producers (Singh et al., 2023). It is difficult to determine whether the overall production of SCFAs was altered or not in the FV group without a metabolomic analysis of the stool samples. However, we speculate that production of SCFAs in the FV group may not have been significantly affected, given that the taxa enriched at both time points were capable of producing SCFAs.

LEfSe analysis also revealed that there was an enrichment in bacterial taxa in the *Lachnospiraceae* family in the FV group at the end of eight weeks. The *Lachnospiraceae* family is part of the clostridial cluster XIVa of the phylum Firmicutes. The increase in taxa from the *Lachnospiraceae* family is supported by two other feeding studies. One study reported an increase in *Lachnospira* in participants consuming high-fibre diets (Wastyk et al., 2021), and another study found an association between *Lachnospira* and high dietary fibre intake (Lin et al., 2018). Bacterial taxa within the *Lachnospiraceae* family form a core part of the microbiome and are known SCFA producers (Vacca et al., 2020). These bacteria colonize the gut during infancy and increase in abundance throughout adulthood. There are studies showing both positive and negative health effects related to *Lachnospiraceae* taxa (Vacca et al., 2020), but there are limited human studies linked to cardiovascular health. One of the few large cohort studies to explore the effect of fermented plants on the gut microbiome used participants enrolled in the American Gut Project (AGP) (Taylor et al., 2020). In this 4-week longitudinal study, 115 individuals in the AGP were enrolled to investigate if regular consumption of fermented plant foods had an impact on the diversity of the gut microbiome (Taylor et al., 2020). No significant differences in alpha diversity were seen between the two groups; however, researchers noted increased levels of conjugated linoleic acid (CLA) in the fermented plant food consumers. CLA has been purported to have many health benefits, including positive effects on atherosclerosis through improvement in the blood lipid profile (Dilzer and Park, 2012; Koba and Yanagita, 2014). In a pilot study investigating the effect of consumption of 100 g of FVs per day, there was an increase in *Faecalibacterium prausnitzii (P* = .022) and a trend towards a decrease in *Ruminococcus torques* (*P* = .074) in healthy female participants after six weeks of the intervention (Galena et al., 2022). A study in South Korea investigated the effect of the consumption of fermented kimchi on obesity (Han et al., 2015). Study participants were assigned to either the fresh (n = 12) or fermented (n = 11) kimchi group for eight weeks, and they consumed 300 grams of kimchi per day (100 grams per meal). Findings showed an increase in *Bacteroides* and *Prevotella* and a decrease in *Blautia* abundance in the fermented kimchi group (Han et al., 2015).

Although our study did not show an effect on alpha diversity, a previous study with a higher dose and duration reported an increase in alpha diversity in healthy adults (Wastyk et al., 2021). In this prospective study, fermented food intake steadily increased over the course of 10 weeks. Participants consumed an average of 0.4 servings at baseline and an average of 6.3 servings at week 10. A wide variety of fermented foods were consumed, which included FVs, yoghurt, kombucha, and kefir (Wastyk et al., 2021). Furthermore, a correlation was found between the number of servings of fermented food and an increase in diversity (Wastyk et al., 2021). In the present study, there were significant differences in beta diversity between the two groups at both time points. It is possible that the high interindividual variability generally reported in the composition of the gut bacteria is responsible for these significant differences between the two groups. Dietary intake was assessed at both time points and there were no significant differences were found in macronutrient intake and intake of other nutrients of interest in the context of CVD, such as sodium, fibre, cholesterol, alcohol, and choline. Notwithstanding, it should also be noted that the method used to assess dietary intake was not sufficiently precise to account for variability in individual dietary intake.

### Effects on cardiovascular risk factors

Participants had at least one risk factor for CVD (BMI > 25, controlled hypertension, or a family history of heart disease). Over the course of the eight weeks, no significant change in weight, percent body fat, or blood pressure was observed. Previous studies have shown an impact on risk factors for CVD. Researchers in a kimchi feeding study in South Korea studied 22 overweight and obese participants (Kim *et al*., 2011). The observations included a small reduction in BMI and body weight in participants who consumed fermented kimchi (Kim *et al*., 2011). In this crossover design study, participants were given both fermented kimchi and fresh kimchi for two four-week periods with a two-week washout phase in between. The amount of kimchi served was 300 grams per day (100 grams per meal), which was three times more than the dose provided in our study. Participants had a small but significant reduction in BMI, body weight, total cholesterol, fasting insulin, and blood glucose levels compared with baseline (Kim *et al*., 2011). A different study investigating the effect of consumption of fermented kimchi on obesity in South Korean women reported improvements in waist-hip ratio, blood pressure, insulin, and fasting blood glucose in participants who consumed 300 *g* of FVs per day (Han *et al*., 2015). Participants were between 30 and 60 years of age, with a BMI >25 and participants consumed 300 grams of kimchi per day (100 grams per meal). Participants in both groups had decreases in body weight, BMI, and body fat, and the fermented kimchi group showed a significant decrease in the waist-hip ratio (Han et al., 2015). These findings indicate that higher doses of FVs (300 *g* per day) in studies conducted in South Korea, where kimchi is commonly consumed, resulted in a significant decrease in body weight. In our study, we used a lower dose of 100 g per day for at least five days per week. While we considered increasing the dose to enhance the effect, most of our participants were not accustomed to consuming FVs regularly, which posed challenges to compliance to the treatment.

### Study strengths and limitations

One limitation of this study is that the results may not be generalizable to the whole population as the majority of study participants were female. In addition, over 80% of the participants were white. Furthermore, we utilized volunteer sampling, and even though study participants were recruited through many methods, including flyers, social media, and mailing lists, it is possible that those interested in participating in the present study were more likely to follow a healthier lifestyle. When it comes to the study design and selection of the control group, it is possible that including 100 g of fresh cabbage as part of the control group treatment would have been a more appropriate comparison group. The FVs consumed during the study added up to 3–4 g of fibre each day; however, based on the nutrient intake comparisons between the two groups, we did not find any significant differences in fibre intake, which indicates that the addition of 100 of FVs to participants’ diets did not result in an increase in fibre intake. In fact, it is more likely that participants in the FV group substituted FVs for another vegetable that was part of their regular diet. Another limitation of this study is that measurement of microbial metabolites would likely have provided additional insight into the effect of consumption of FVs on microbial metabolism in the gut. Our laboratory has recently acquired funding to explore the role of FVs on microbial metabolites in stool samples, which will provide a more complete account of how FVs may modulate gut microbial metabolism. A unique aspect of the present study was the large amount of data collected: assessment of stool, body measurements, and blood biomarkers combined with dietary surveys, gastrointestinal symptom collection surveys, and physical activity assessment. Also, statistical power was strengthened by the stool collection protocol, which allowed for the analysis of three separate stool samples collected in three consecutive days at both time points for a total of six samples per participant. We also conducted a feasibility survey at the end of the study period to collect information on the participants’ reactions to being in the FV group. Most participants reported that they had no difficulty consuming the FVs in the frequency and amount required. Many participants reported that they would continue to consume FVs on at least a weekly basis after the study period, indicating that it is possible to incorporate FVs into the regular diet of Americans.

In conclusion, consuming 100 grams of FVs at least five days per week for eight weeks did not change the levels of inflammatory biomarkers or alpha diversity based on Shannon index measures. However, discriminant analysis showed a greater number of alterations in the FV group compared with the UD group.

### Future directions

Understanding the symbiosis of the human diet and the microbial composition of the gut, including metabolites, is important for future research. In general, because there have been so few feeding studies, more of these types of studies are needed, particularly with a greater length of time and a larger quantity and variety of fermented foods for a possible greater effect. Furthermore, future studies should consider the role fermented foods play in bowel health and regularity. In our study, participants in the FV group reported a greater bowel movement frequency of 1.9 times per day, whereas participants in the UD group reported less than one bowel movement daily (0.8/day). Participants in the FV group reported positive changes in their bowel movements, particularly improved regularity, less malodorous stool, and a notable reduction in hard stools and constipation, although these data were not systematically captured during the present study. These results indicate a potential link between a reduction in constipation and FV intake that should be further investigated. Another area that requires further investigation is the identification of individuals who may or may not benefit the most from the regular consumption of FVs. Studies focussing on individuals with low or high levels of inflammatory markers might also provide additional insight into commonalities and differences in the microbiota, especially as it relates to protection against CVD.

## Supporting information

Baron et al. supplementary material Baron et al. supplementary material

## Data Availability

The data that support the findings of this study are openly available in Dryad at https://doi.org/10.5061/dryad.547d7wmfk

## Supplementary material

The supplementary material for this article can be found at http://doi.org/10.1017/gmb.2024.4.

## Social media summary

New study finds that daily consumption of half a cup of fermented vegetables for two months is not sufficient to alter inflammation markers or the diversity of the gut bacteria in adults at risk for cardiovascular disease.
