# Supplementary figures and images for: The effects of fermented vegetables on the gut microbiota for prevention of cardiovascular disease
Source: Gut Microbiome (Camb). 2024 May 9;5:e6. doi: 10.1017/gmb.2024.4 (PMC11404656; doi:10.1017/gmb.2024.4)

**A**

weighted Unifrac

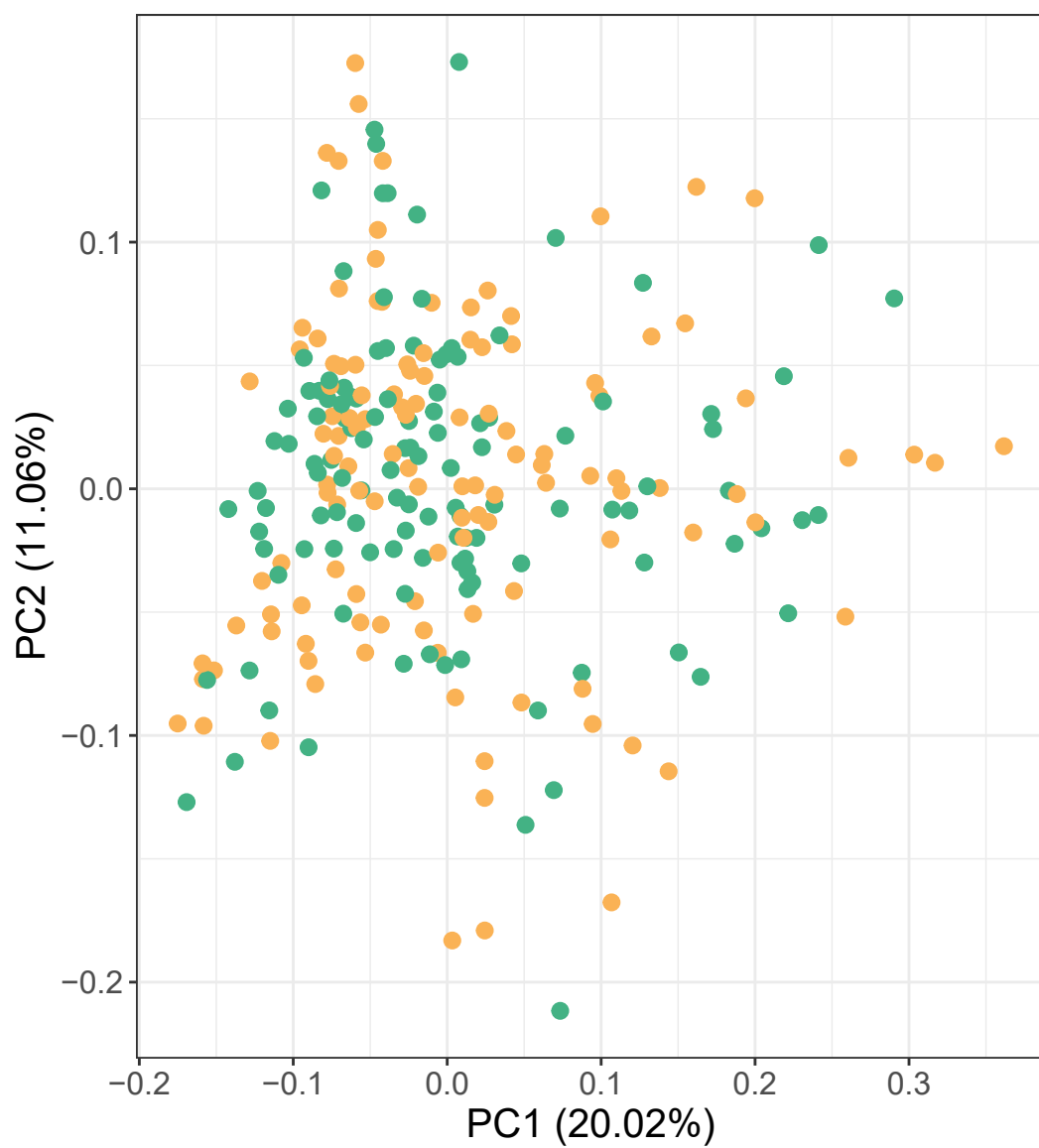**B**

unweighted Unifrac

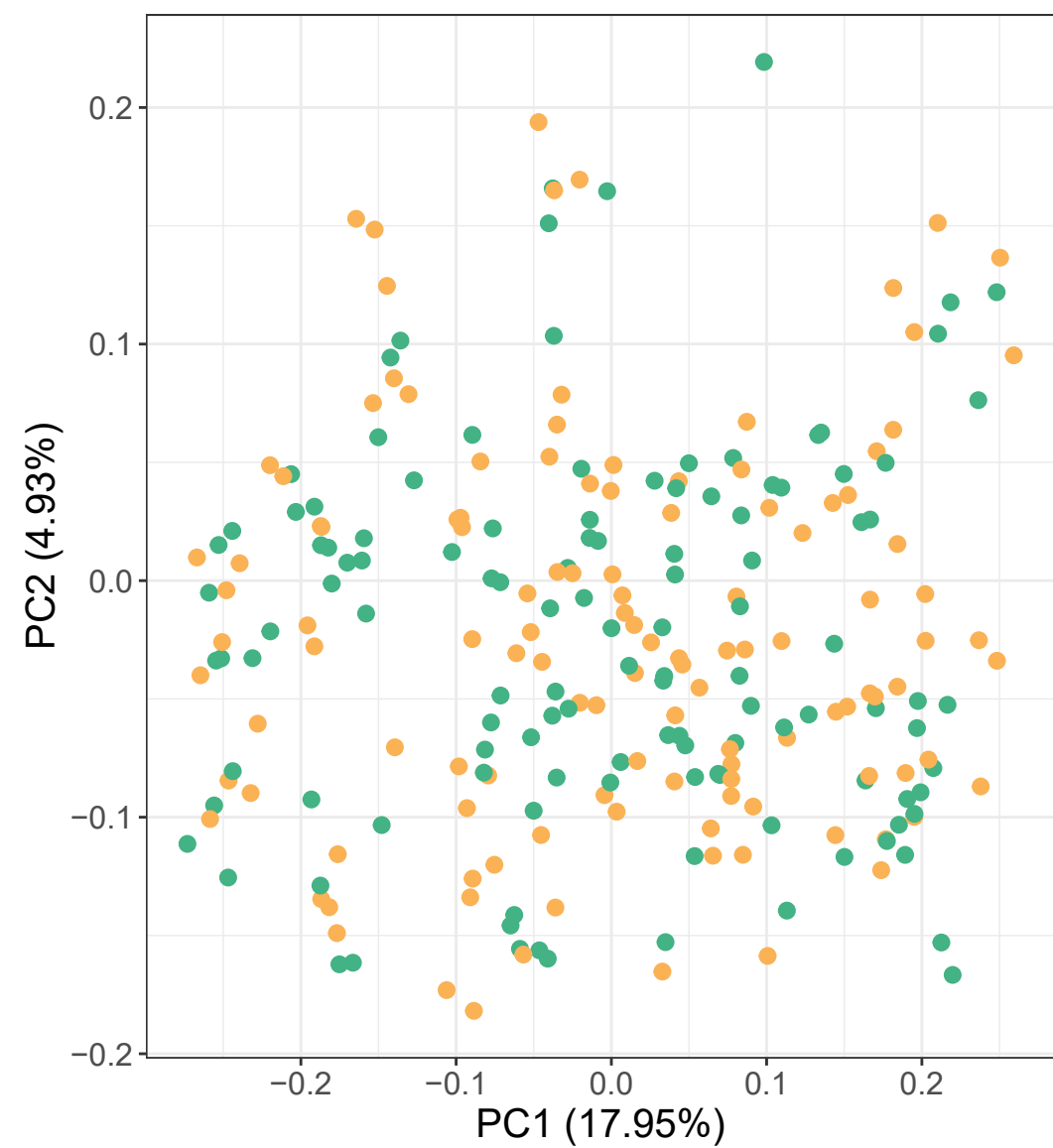**C**

weighted Unifrac

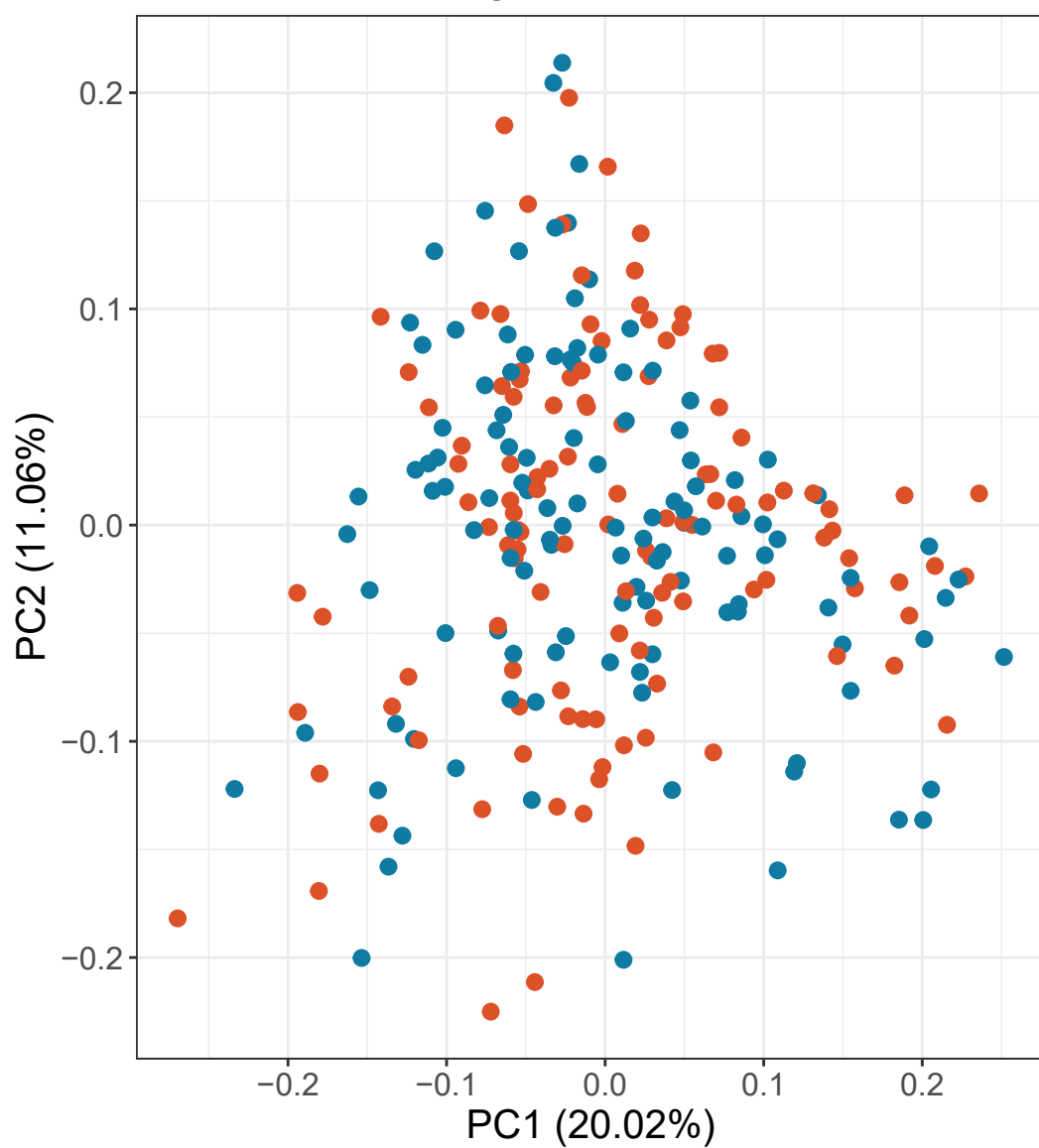**D**

unweighted Unifrac

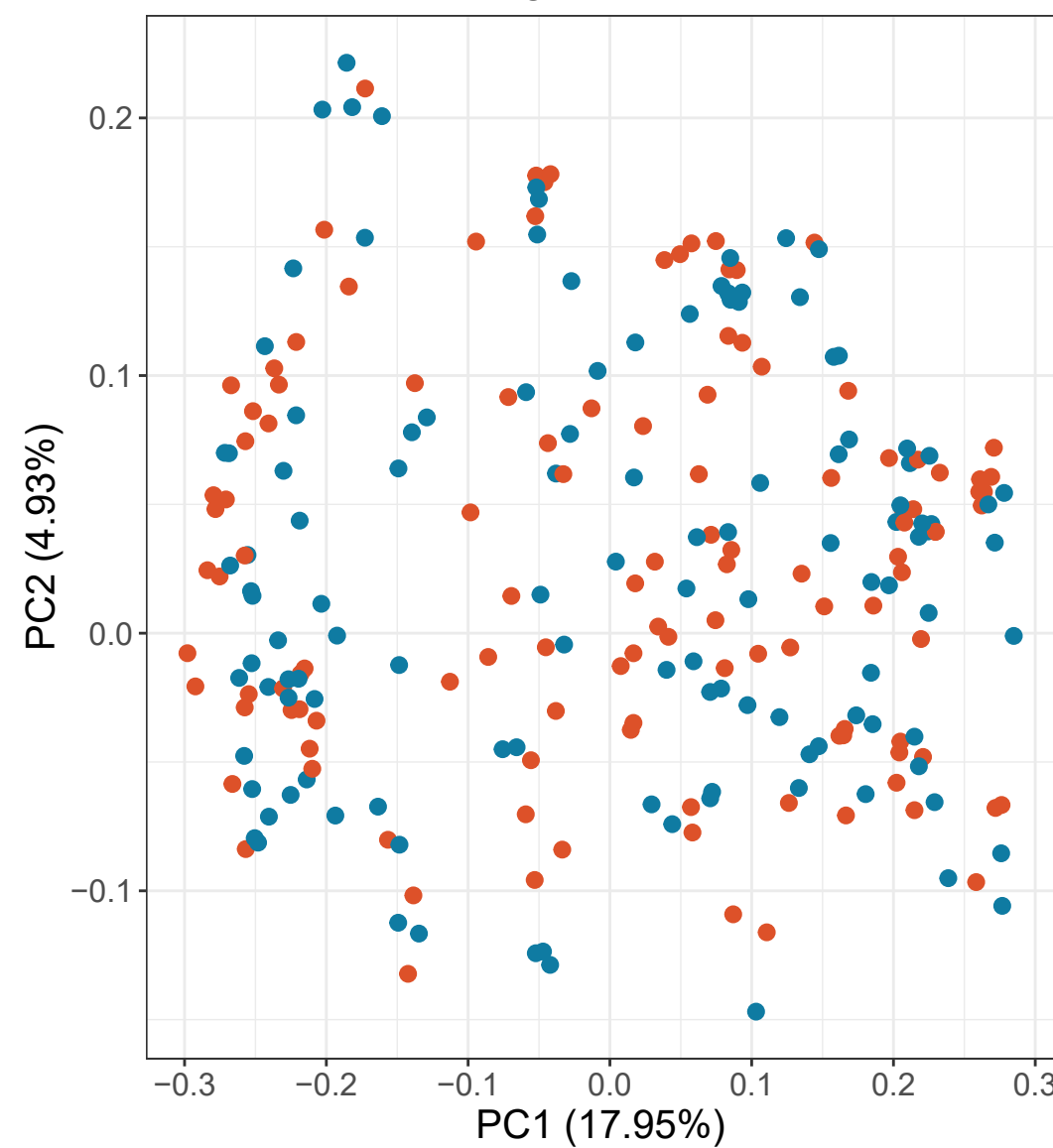

Supplement: Baron et al. supplementary material [file S2632289724000045sup001.pdf]
